# Concise stakeholder-informed tool for reporting modifications to innovative surgical procedures and devices: Surgical ModificAtion Reporting Tool (SMART) checklist

**DOI:** 10.1093/bjs/znag117

**Published:** 2026-10-05

**Authors:** James B Olivier, Rhiannon C Macefield, Maeve Coyle, Kerry N L Avery, Natalie S Blencowe, Daisy Elliott

**Affiliations:** NIHR Bristol Biomedical Research Centre, University Hospitals Bristol and Weston NHS Foundation Trust and University of Bristol, Bristol, UK; NIHR Bristol Biomedical Research Centre, University Hospitals Bristol and Weston NHS Foundation Trust and University of Bristol, Bristol, UK; NIHR Bristol Biomedical Research Centre, University Hospitals Bristol and Weston NHS Foundation Trust and University of Bristol, Bristol, UK; NIHR Bristol Biomedical Research Centre, University Hospitals Bristol and Weston NHS Foundation Trust and University of Bristol, Bristol, UK; NIHR Bristol Biomedical Research Centre, University Hospitals Bristol and Weston NHS Foundation Trust and University of Bristol, Bristol, UK; Leeds Institute of Emergency General Surgery, St James’s University Hospital, Leeds, UK; NIHR Bristol Biomedical Research Centre, University Hospitals Bristol and Weston NHS Foundation Trust and University of Bristol, Bristol, UK

## Abstract

**Background:**

Modifications to invasive procedures and devices are common during surgical innovation, yet reporting is frequently inconsistent and incomplete. The aim of this study was to develop a concise, stakeholder-informed checklist for reporting modifications in surgical innovation studies.

**Methods:**

A provisional checklist was developed from a systematic review of 104 IDEAL (Idea, Development, Exploration, Assessment, and Long-term study) studies, identifying substantial heterogeneity and deficits in modification reporting. Twenty stakeholders were purposively sampled for semi-structured interviews, including clinicians, methodologists, industry representatives, and governance professionals. Interviews explored views and subsequently optimized the checklist’s content, wording, layout, scope, and usability. Data were analysed by thematic analysis and iteratively used to refine the checklist until no further required changes were identified.

**Results:**

Stakeholders endorsed the need for a dedicated modification reporting tool, emphasizing pragmatism and usability. Interview findings led to clarification of scope, refinement of terminology, and removal of ambiguous items. Greater emphasis was placed on describing the innovation’s starting configuration, specifying the IDEAL stage, explicitly reporting both the presence and absence of modifications, and reflecting on procedural stability. Iterations condensed the checklist to a seven-item tool. The final Surgical ModificAtion Reporting Tool (SMART) specifies describing the starting point of the innovation, reporting modifications, and summarizing stability and shared learning.

**Conclusion:**

SMART is a concise, stakeholder-informed prepublication tool to guide prospective tracking and consistent reporting of modifications to innovative invasive procedures and devices. Used alongside the IDEAL framework, it has the potential to improve the quality and interpretability of surgical innovation research.

## Teaser

This study developed the Surgical ModificAtion Reporting Tool (SMART), a concise seven-item checklist designed to standardize the reporting of modifications to innovative surgical procedures and devices. Refined through a systematic review and 20 stakeholder interviews, the tool focuses on documenting the innovation’s starting configuration, the rationale for modifications, and overall procedural stability. Intended to complement the IDEAL framework, SMART aims to enhance transparency, safety, and shared learning across the surgical innovation community.

## Introduction

Surgical innovation is central to improving patient outcomes, yet, historically, the introduction of new procedures and devices has been largely unstructured and empirical^1^. Innovations characteristically evolve through iterative modifications as surgeons accumulate experience and respond to early outcomes^2,3^. The IDEAL (Idea, Development, Exploration, Assessment, and Long-term study) and IDEAL-D (IDEAL for Devices) frameworks were developed to address this, providing a staged structure for evaluating surgical innovations^4,5^. In practice, however, innovation development is often cyclical rather than strictly sequential, and modifications are an inherent and defining feature of this process, particularly during the early stages^1^. Transparent reporting of these modifications is therefore fundamental to the safe and effective evaluation of surgical innovation.

When modifications are unreported or inadequately described, consequences can be serious^6^. If undocumented and unshared, beneficial modifications remain siloed within individual centres, slowing diffusion and hindering collaborative learning^6^. Similarly, detrimental modifications may be unknowingly repeated, contributing to research waste and, most critically, patient harm^7^. Poor modification reporting also complicates governance decisions. Without clear documentation of what has changed and when, it becomes difficult for institutional committees and regulators to distinguish minor refinements from substantively new interventions requiring formal oversight^8^. The absence of a standardized definition and reporting approach for modifications has been identified as a significant gap in current surgical governance frameworks^6,7,9^.

Structured reporting tools monitored by the EQUATOR (Enhancing the QUAlity and Transparency Of health Research) Network, such as CONSORT, PRISMA, IDEAL, and SCARE, have systematically enhanced research transparency and reduced reporting bias across diverse clinical designs^10–19^. When endorsed or mandated by surgical journals, concise checklists regularly become embedded in routine research culture and improve reporting standards^11,20,21^. Yet, despite these advances, no equivalent tool exists to guide the reporting of modifications in surgical innovation, a gap with direct implications for patient safety and research transparency^7^.

Whilst IDEAL acknowledges the importance of reporting modifications, particularly during early development, it does not provide detailed guidance on how to do so^19,22^. The COHESIVE (Core Outcomes for Early Phase Surgical Innovation and Devices) Core Outcome Set (COHESIVE-COS) identifies modification reporting as a core domain, but similarly stops short of specifying what information authors should include or how it should be presented^23,24^. A scoping review of secondary literature confirmed a lack of consensus on how modifications are defined, classified, or reported, and emphasized the need for a standardized approach to facilitate shared learning^7^.

This paper describes the development and finalization of the Surgical ModificAtion Reporting Tool (SMART), a concise, stakeholder-informed checklist to support consistent reporting of modifications to innovative invasive procedures and devices.

## Methods

### Study design

A mixed-methods study was conducted in three phases. Phase 1 involved a systematic review of IDEAL and IDEAL-D studies to characterize current modification reporting practice and generate potential items, wording, and domains for inclusion in the reporting checklist. Phase 2 included the development and formatting of a provisional checklist. Phase 3 utilized cognitive stakeholder interviews to iteratively refine and finalize the checklist. Phase 1 has been published in full elsewhere^25^ and is summarized alongside phase 2 in *Appendix S1* to contextualize checklist development. The present study focuses on phase 3 and is reported in accordance with the Standards for Reporting Qualitative Research (SRQR)^26^. A completed SRQR checklist is provided in *Appendix S2*.

### Registration

The systematic review protocol^25^ was prospectively registered on the PROSPERO database (registration number CRD42023427704; https://www.crd.york.ac.uk/prospero/display_record.php?ID=CRD42023427704). The protocol for the qualitative phase and checklist development was not preregistered.

### Participants and sampling

Ethical approval was granted by the University of Bristol Faculty of Health Sciences Research Ethics Committee (Reference ID: 56522). Participants were purposively sampled to represent a range of perspectives relevant to surgical innovation and its evaluation^27,28^. Eligible participants included clinicians, methodologists, industry representatives, and governance and regulatory professionals, all with direct experience in developing innovative surgical procedures and/or devices. This direct experience was defined as active professional involvement in the clinical delivery, technical design, methodological evaluation, or regulatory oversight of a novel procedure or device. As phase 3 tested the operational utility of a prepublication tool, the interview participants focused on ‘target users’; those who would complete or review the checklist. Initial participants were identified through established collaborative research networks, specifically the National Institute for Health and Care Research (NIHR) Bristol Biomedical Research Centre and clinical investigator cohorts from prior surgical innovation evaluation studies^24,29^. Snowball sampling was then used, whereby participants suggested further individuals with relevant expertise^30^. Recruitment was guided by a purposive sampling framework established at the outset of the study to ensure the inclusion of a diverse range of professional perspectives and clinical experience. Participant characteristics were continuously monitored throughout the recruitment process, allowing subsequent iterations of sampling to actively target specific stakeholder groups and clinical specialties whose views had not yet been sufficiently represented^30^. To ensure adequate representation across stakeholder groups, sampling continued until data saturation was achieved, defined as when consecutive interviews within a group yielded no new themes or checklist changes^31,32^.

### Data collection

Cognitive interviewing involves participants verbalizing their thought processes in real time as they review a document, which is supplemented by targeted verbal prompts from the interviewer^33,34^. Cognitive interviews were used to observe stakeholders interacting directly with the provisional checklist, thereby capturing immediate reactions to item wording, layout, and usability. By exploring how specific items are understood, this approach identifies hidden ambiguities and misinterpretations, ensuring the tool is interpreted consistently before final implementation^31,33^. Interviews were conducted one-to-one to elicit individual perspectives that might not emerge in a group setting^31^. Participants were sent a participant information letter and informed consent form before their interviews. Consent was verbally confirmed before commencing each interview.

All interviews were conducted by the lead author (J.B.O.), a surgical trainee with training in qualitative methodology and interviewing. A pilot interview was conducted with a study team member before recruitment of external participants. An interview topic guide was used to explore the perceived need, scope, clarity, wording, layout, length, and usability of the checklist, as well as potential applications beyond journal publication, including governance documentation and registries. The topic guide was refined iteratively throughout data collection to allow for exploration of new themes as they emerged. The provisional checklist was shared with participants during the interview rather than in advance, to replicate real-world usage conditions and capture immediate responses.

Interviews were audio-recorded and transcribed verbatim. Regular team reflexivity discussions were held throughout to consider how members’ professional backgrounds and perspectives may have influenced data collection and interpretation^35^.

### Data analysis

Transcripts were analysed using a hybrid deductive and inductive approach through thematic analysis^36,37^, underpinned by a critical realist paradigm^38,39^. An initial coding framework was mapped deductively using the items from the provisional checklist (phase 2) as a baseline, while leaving the analytical process sufficiently flexible to capture newly emergent, inductive themes directly from the stakeholder data^40^. Analysis was performed by J.B.O. using qualitative software (NVivo 14)^41^. This was done in batches so that refinements could be made to the checklist after each group of interviews. A subset of transcripts was independently reviewed by a second team member (D.E.) to enhance credibility and add breadth to the analysis^42^. Codes captured participants’ views on the importance, clarity, and practicality of items, suggestions for additions, removals, or rewording, and views on layout and scope. Codes were grouped into themes and subthemes, refined iteratively through study team discussion.

### Checklist refinement

Checklist refinement proceeded concurrently with data collection and analysis. After each group of interviews, the team reviewed emerging themes and their implications for the checklist. Changes included merging or splitting items, revising wording and terminology, reordering items for logical flow, and adjusting layout and overall length. A version log recorded all iterations and their corresponding rationales. Data collection ceased when the study team agreed that no new major themes or substantive checklist changes were being identified. An independent researcher experienced in qualitative methodology (M.C.) reviewed the final three transcripts to confirm that data saturation appeared to have been reached.

## Results

Similar to the Methods section of this paper, the Results section places focus on reporting phase 3 of this research (stakeholder interviews) and the resulting SMART checklist finalization.

### Participants and interviews

Semi-structured cognitive interviews were conducted between July and November 2024. Of 34 individuals invited, 24 expressed interest, and 20 ultimately participated. Interviews lasted a median of 34 (range 16–81) min; 19 were conducted virtually and 1 was conducted face-to-face. Participants included clinicians (15 participants), trialists or methodologists (8 participants), industry representatives (2 participants), and governance professionals (2 participants), with several participants having more than one type of profession (*Table 1*). Sixteen participants were male, and four were female, from ten UK regions. Most clinicians were consultants, representing a range of surgical and medical specialties. Eight of the participants had formal roles within medical journals, including Editor-in-Chief, Assistant Editor, and Editorial Board positions.

**Table 1:** Summary of participant characteristics

Characteristic	Classification	Number of participants (*n* = 20)
Sex	Male	16
	Female	4
Title/role*	Professor/Associate Professor	10
	Consultant	5
	Resident Doctor	2
	Other	3
Type of profession*	Clinician	15
	Methodologist	8
	Industry	2
	Governance	2
	Editorial role	8
Length of time in main role (years)	0–4	2
	5–9	11
	>9	6
	Retired	1
Specialty	Colorectal	3
	Trauma and orthopaedics	2
	Plastic surgery	2
	Hepatopancreatobiliary (HPB) surgery	1
	Bariatric surgery	1
	Ophthalmic surgery	1
	Urology	1
	Emergency general surgery	1
	Neurosurgery	1
	Obstetrics and gynaecology	1
	Clinical physiologist	1
	Renal medicine	1
	Non-clinical—statisticians	2
	Non-clinical—engineers	2

^*^Categories are not mutually exclusive; participants could have more than one type of profession.

### Themes

Analysis of interview data generated four overarching themes that informed refinement of the checklist: perceived need and scope; clarity and content of items; length and layout; and essential components for modification reporting. Illustrative quotations are presented in *Table S1*.

### Perceived need and scope

Stakeholders consistently endorsed the need for a dedicated modification reporting tool, recognizing a clear gap in existing guidance and expressing confidence that the checklist would be adopted in practice as a prepublication standard. The potential scope of the tool beyond academic publication was also discussed. Participants identified governance documentation and registry reporting as settings where structured modification reporting would be of value, and suggested that item wording should remain broad enough to accommodate these uses.

### Clarity and content of items

Participants considered most items relevant but suggested several refinements. Terminology relating to patient selection was noted to vary across the literature; ‘patient eligibility criteria’ was preferred over ‘patient selection criteria’ as clearer and more patient-centred. The term ‘non-technical modifications’ was perceived as ambiguous and difficult to operationalize; participants differed on whether it should encompass contextual factors, team composition, or broader service changes. Items referencing non-technical modifications were therefore removed. Stakeholders strongly endorsed items requiring authors to describe the innovation’s starting configuration and to specify the applicable IDEAL or IDEAL-D stage, to contextualize modification reporting within the framework.

### Length and layout

Usability and pragmatism emerged as a prominent theme. Participants expressed concern that lengthy or complex checklists discourage uptake, citing examples of other reporting tools perceived as onerous. They advocated for a concise, single-page format with actionable, understandable item wording. In response, overlapping items were merged or condensed, and the checklist was reduced from ten to seven items. The layout was redesigned to fit on a single A4 page. Participants favoured a format aligned with established reporting guidelines; the tick-box column was replaced with a ‘page/section number’ field, enabling authors and reviewers to indicate where each item is addressed in the manuscript.

### Essential components for modification reporting

Interviews reinforced and extended several components regarded as essential. Participants emphasized the need to document what changed (for example, procedural steps, device design, or components), when in the series the change occurred (for example, by patient number or date), and why the modification was introduced, including observed benefits and drawbacks. The explicit reporting of the absence of modifications was identified as particularly important; participants noted that this is rarely stated but is critical for interpreting claims of innovation stability. Graphical timelines of modifications were regarded as helpful, particularly in studies where innovations evolved significantly, as they enable readers to link changes to outcomes or complications. Finally, participants valued a dedicated prompt to summarize key learning points from the modification process in the discussion, to support knowledge transfer and inform future adoption or adaptation of the innovation.

### Checklist finalization and structure

Informed by these findings, the checklist was condensed and finalized as the SMART checklist (*Fig. 1*). The seven items were grouped into three domains: domain 1—describe the starting point of the innovation; domain 2—report modifications; and domain 3—reflecting on modifications. A timeline of the checklist iterations/versions across the 20 interviews is provided as *Fig. S1*, and a hypothetical example of a completed checklist to demonstrate the practical application of the tool is provided for illustrative purposes in *Fig. S2*.

**Figure 1:**
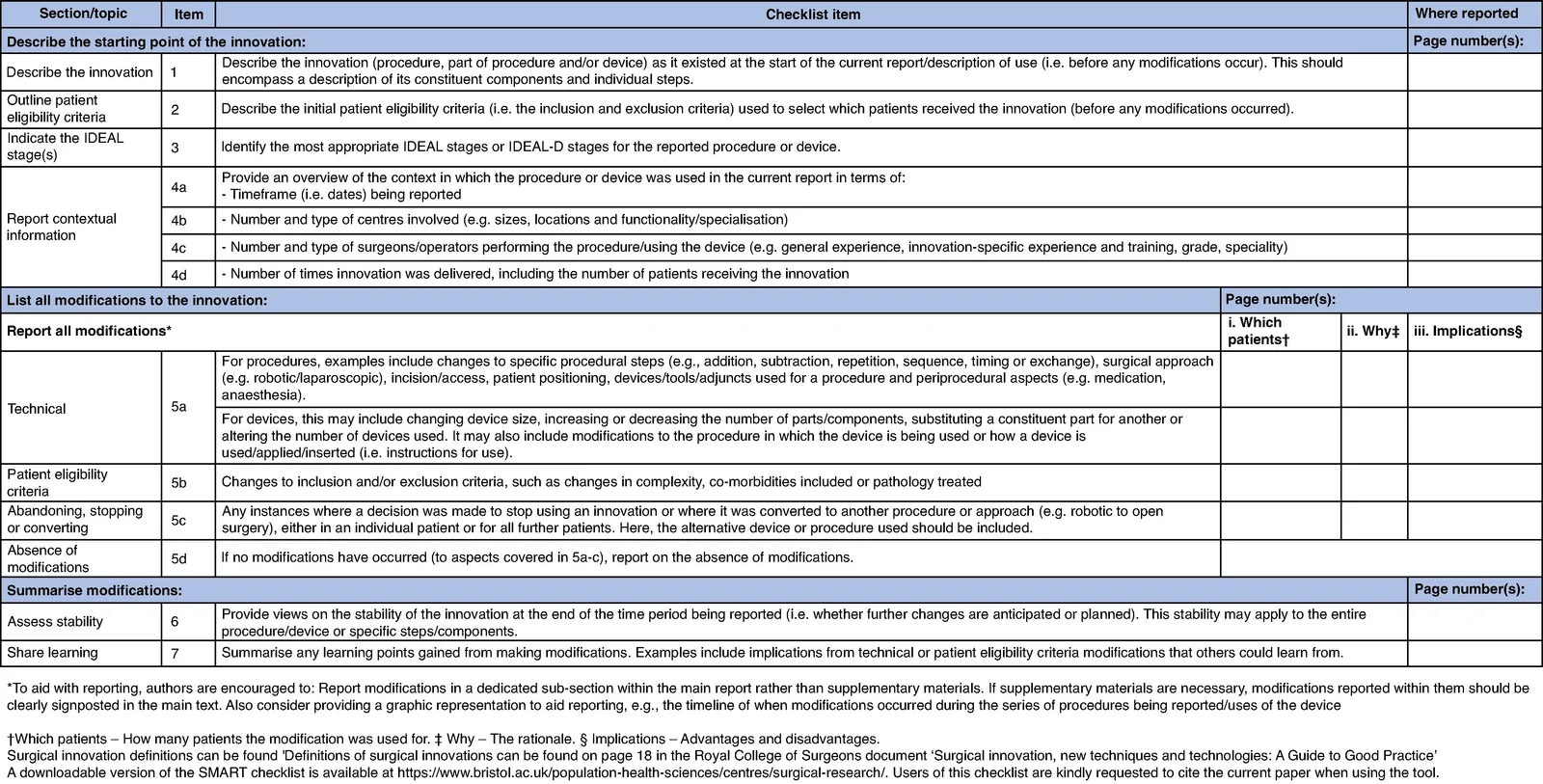
Final version of SMART—a checklist for reporting modifications to an innovative procedure or device SMART, Surgical ModificAtion Reporting Tool.

### Domain 1: describe the starting point of the innovation (items 1–4d)

This domain establishes a baseline control state for the innovation before modifications occur. It mandates describing the intervention’s initial technical configuration (item 1) and premodification patient eligibility criteria (item 2). Authors must also declare their corresponding evaluation stage within the IDEAL or IDEAL-D framework (item 3). Environmental context must be quantified across four areas: time frame (item 4a), institutional settings (item 4b); innovation users (item 4c); and cumulative delivery volumes regarding both procedures performed and individual people treated (item 4d).

### Domain 2: report modifications (items 5a–d)

The second domain standardizes modification reporting using an internal matrix covering which patients, why (the rationale), and implications (advantages and disadvantages). This applies directly to procedural or device changes (item 5a), alterations to patient eligibility criteria (item 5b), and modifications involving converting, pausing, or abandoning the innovation (item 5c). To facilitate transparent reporting, a definitive statement confirming the absolute absence of modifications is required if no adjustments occurred (item 5d).

### Domain 3: reflecting on modifications (items 6 and 7)

The final domain moves from descriptive parameters to reflective synthesis. Authors must comment on the maturity and stability of the innovation at the study’s conclusion, clarifying whether it has reached standardization or requires continuing iterative development (item 6). Finally, authors are required to share explicit take-home learning points to inform external institutional adaptation, enhance patient safety, and prevent downstream research waste (item 7).

## Discussion

This study reports the development, refinement, and finalization of the SMART checklist, a concise tool to support the reporting of modifications to innovative invasive procedures and devices. SMART is a streamlined, seven-item checklist describing the starting point of the innovation and its context, with structured reporting of what changed, when it changed, and why it changed, as well as reflection on learning and innovation stability. By enabling more transparent and structured documentation of how an innovation evolves, SMART has the potential to support safer diffusion of beneficial changes and reduce the risk of unreported or repeated harmful modifications.

The SMART checklist is intended to complement, rather than replace, IDEAL and IDEAL-D. IDEAL provides a staged framework for evaluating surgical innovation^4^, and recent work has emphasized the need for better reporting of procedural and device modifications, particularly in the early stages of their development and use^7^. SMART operationalizes the modification domain of the COHESIVE-COS, which recommends that modifications to procedures, devices, and patient selection criteria be reported in all surgical innovation studies^23,24^, by specifying what information authors should report about modifications and how it can be presented. In this respect, it is analogous to tools developed for core outcome sets and reporting guidelines, which aim to standardize key elements of research reporting to reduce heterogeneity and facilitate synthesis^43,44^. Its focus on succinct item wording and single-page layout reflects lessons from other tools about the importance of usability for routine adoption^44,45^.

Although the SMART checklist has been developed to support investigators to report modifications at the conclusion of a patient series or during manuscript preparation, this necessarily requires consideration at study inception, to facilitate rigorous, progressive data collection in real time. Ideally, researchers should document modifications as they occur within the clinical workflow, for instance after each individual procedure, to ensure a reliable record is available at the end of the series. Relying on retrospective recall at the point of manuscript submission may introduce recall bias and selective reporting. To maximize data integrity, these parameters could be recorded within routine operative logs or case report forms from research studies.

This study has several strengths. The systematic review characterized current modification reporting practice across a large number of IDEAL and IDEAL-D studies, ensuring that the checklist reflects real-world challenges and examples of good practice. The qualitative phase involved multiple stakeholder groups, helping to ensure relevance across perspectives and potential uses. Iterative refinement continued until no new major suggestions emerged, supporting the credibility of the final version. Study limitations include the restriction to UK-based participants and the use of semi-structured interviews rather than a formal international Delphi consensus process. While a Delphi survey can quantify agreement across a larger international panel^46^, cognitive interviewing was selected to allow an in-depth evaluation of how target users understood and practically applied each checklist item. Additionally, the reliance on snowball sampling introduces a potential risk of network bias, where participants may recommend peers with similar professional perspectives. The present study sought to minimize this risk by utilizing diverse initial networks and intentionally seeking maximal variation in participant recruitment across ten geographical regions and 12 separate clinical fields. Future validation work in international settings could complement this approach by testing checklist uptake and generalizability across different healthcare systems. Finally, the checklist has not yet been prospectively evaluated in ongoing innovation studies. Nonetheless, given the current variability in modification reporting across the surgical innovation literature, the development of SMART represents an important step towards improving transparency and consistency in the reporting of surgical innovation.

SMART offers clinicians and researchers a practical structure for documenting how an innovation changes over time, making it easier for readers, reviewers, and regulators to interpret outcomes and complications within the context of innovation evolution^47^. Future work should evaluate the feasibility of using SMART prospectively in case studies and assess whether its implementation improves the completeness of modification reporting. As the IDEAL framework extends into adjacent fields, including physiotherapy (IDEAL-Physio), artificial intelligence (DECIDE-AI), and robotics (IDEAL Robotics), there is also significant scope to explore whether SMART can support modification reporting within these specialized disciplines^48–52^. Ultimately, by providing concise, stakeholder-informed guidance, the SMART checklist has clear potential to enhance the transparency, interpretability, and reproducibility of surgical innovation research internationally.

## Supplementary Material

znag117_Supplementary_Data

## Data Availability

Anonymized interview transcripts are not publicly available due to the risk of participant identification. A downloadable version of the SMART checklist is freely available via the University of Bristol Centre for Surgical Research web page: https://www.bristol.ac.uk/population-health-sciences/centres/surgical-research/. Use of the checklist must cite this publication.
