# Supplementary material for: Concise stakeholder-informed tool for reporting modifications to innovative surgical procedures and devices: Surgical ModificAtion Reporting Tool (SMART) checklist
Source: Br J Surg. 2026 Oct 5;113(10):znag117. doi: 10.1093/bjs/znag117 (PMC13637718; doi:10.1093/bjs/znag117)
Supplement: Supplementary file 1 [file znag117_supplementary_data.docx]

**A concise stakeholder-informed tool for reporting modifications to innovative surgical procedures and devices: the SMART checklist**

James B Olivier*^1^, Rhiannon C Macefield^1^, Maeve Coyle^1^, Kerry N.L. Avery^1^, Natalie S Blencowe^1,2^, Daisy Elliott^1^

1 - NIHR Bristol Biomedical Research Centre, University Hospitals Bristol and Weston NHS Foundation Trust and University of Bristol, BS8 2BN

2 - Leeds Institute of Emergency General Surgery, St James's University Hospital, Leeds, UK

* - Corresponding author – [James.Olivier@nhs.net](mailto:James.Olivier@nhs.net). ORCID iD 0000-0003-3082-8426. Twitter/X: @jamesolivierSW

| **Supplementary Appendixes** |  |
| --- | --- |
| Supplementary Appendix S1 – Development of the SMART checklist (Methods and Results) | *Pages. 2-3* |
| Supplementary Appendix S2 – Standards for Reporting Qualitative Research (SRQR) | *Pages. 4-5* |
|  |  |
| **Supplementary Figures and Tables** |  |
| Supplementary Table S1 – Illustrative quotes supporting themes identified in stakeholder interviews | *Page 6* |
| Supplementary Figure S1 – Timeline of all 20 interviews with significant checklist version updates and key changes annotated | *Page 7* |
| Supplementary Figure S2 – Illustrative hypothetical example use of SMART (Surgical ModificAtion Reporting Tool) – A checklist for reporting modifications to an innovative procedure or device | *Pages 8-9* |
|  |  |

**Supplementary Appendix S1**

**Development of the SMART checklist (Methods and Results)**

**METHODS**

**Phase 1: Systematic review (content generation)**

A systematic review examined how modifications to innovative invasive procedures and devices are currently identified, described, and contextualised in IDEAL and IDEAL-D studies^1^. Studies were identified using the IDEAL framework as an inclusion criterion because IDEAL-cited studies explicitly engage with methodological guidance on surgical innovation, and were therefore anticipated to provide the broadest and most reflective examples of modification reporting in current practice. Briefly, 104 studies across IDEAL stages 0 to 4 were included. Given the exploratory nature of the review and the absence of a formal definition of ‘modification,’ we extracted all data potentially related to modifications verbatim. This included information about changes to the technical components of the procedure or device, as well as changes relating to patient selection. Data were subsequently analysed by thematic synthesis to assess and learn from current modification reporting practice.

**Phase 2: Provisional checklist development**

Systematic review findings were used to draft a provisional 10-item checklist. Development involved iterative discussion within the research team, comprising clinicians and non-clinical methodologists with expertise in qualitative research, reporting guidelines, and surgical innovation. The development process followed principles established by the EQUATOR Network for developing health research reporting guidelines^2, 3^ and drew on the structure and layout of established checklists, particularly CONSORT, to ensure logical flow and ease of completion^4^. Items were worded to be actionable and understandable to clinicians reporting a surgical innovation study. A key principle guiding item development was to encourage maximum transparency whilst remaining pragmatic. Modifications may range from seemingly minor to major, each with potential implications for patient safety^5^. The checklist, therefore, was designed to prompt reflection on a broad range of change types, including technical modifications to the procedure or device, alterations to patient eligibility criteria, surgeries where the procedure may be abandoned or converted, and, importantly, instances where no modifications were made. Successive drafts were refined by the study team until ready for pre-testing in Phase 3.

**RESULTS**

**Phase 1: Systematic review findings and Phase 2: Provisional Checklist Development**

The previously published systematic review included 104 IDEAL and IDEAL-D studies of innovative procedures and devices across IDEAL stages 0 to 4^1^. Modification reporting varied widely: some studies explicitly identified and described multiple modifications with contextual detail, whilst others reported few or none. Common deficits included incomplete description of the innovation's configuration at the start of the study, inconsistent reporting of when modifications occurred, limited explanation of the rationale for changes or their perceived implications, rare explicit reporting of the absence of modifications, limited use of graphical representations, and few reflections on innovation stability. A provisional 10-item reporting checklist was developed from these findings, grouped into three domains: describing the starting point of the innovation and context, reporting modifications, and presenting and sharing learning.

| **Supplementary Appendix S2**  **Standards for Reporting Qualitative Research (SRQR)*** | |  | |
| --- | --- | --- | --- |
| <http://www.equator-network.org/reporting-guidelines/srqr/> | |  | |
|  | | **Page/line no(s).** | |
| **Title and abstract** |  | |  |
| **Title** - Concise description of the nature and topic of the study Identifying the study as qualitative or indicating the approach (e.g., ethnography, grounded theory) or data collection methods (e.g., interview, focus group) is recommended | | Page 1 | |
| **Abstract** - Summary of key elements of the study using the abstract format of the intended publication; typically includes background, purpose, methods, results, and conclusions | | Page 2 | |
|  | |  | |
| **Introduction** |  | |  |
| **Problem formulation** - Description and significance of the problem/phenomenon studied; review of relevant theory and empirical work; problem statement | | Page 3 | |
| **Purpose or research questio**n - Purpose of the study and specific objectives or questions | | Page 3 | |
|  | |  | |
| **Methods** |  | |  |
| **Qualitative approach and research paradigm** - Qualitative approach (e.g., ethnography, grounded theory, case study, phenomenology, narrative research) and guiding theory if appropriate; identifying the research paradigm (e.g., postpositivist, constructivist/ interpretivist) is also recommended; rationale** | | Pages 4/5 | |
| **Researcher characteristics and reflexivity** - Researchers’ characteristics that may influence the research, including personal attributes, qualifications/experience, relationship with participants, assumptions, and/or presuppositions; potential or actual interaction between researchers’ characteristics and the research questions, approach, methods, results, and/or transferability | | Page 5 | |
| **Context** - Setting/site and salient contextual factors; rationale** | | Pages 4/5 | |
| **Sampling strategy** - How and why research participants, documents, or events were selected; criteria for deciding when no further sampling was necessary (e.g., sampling saturation); rationale** | | Pages 4/5 | |
| **Ethical issues pertaining to human subjects** - Documentation of approval by an appropriate ethics review board and participant consent, or explanation for lack thereof; other confidentiality and data security issues | | Page 4 | |
| **Data collection methods** - Types of data collected; details of data collection procedures including (as appropriate) start and stop dates of data collection and analysis, iterative process, triangulation of sources/methods, and modification of procedures in response to evolving study findings; rationale** | | Page 5 | |
| **Data collection instruments and technologies** - Description of instruments (e.g., interview guides, questionnaires) and devices (e.g., audio recorders) used for data collection; if/how the instrument(s) changed over the course of the study | | Pages 4/5 | |
| **Units of study** - Number and relevant characteristics of participants, documents, or events included in the study; level of participation (could be reported in results) | | Pages 6/7 | |
| **Data processing** - Methods for processing data prior to and during analysis, including transcription, data entry, data management and security, verification of data integrity, data coding, and anonymization/de-identification of excerpts | | Page 5 | |
| **Data analysis** - Process by which inferences, themes, etc., were identified and developed, including the researchers involved in data analysis; usually references a specific paradigm or approach; rationale** | | Page 5 | |
| **Techniques to enhance trustworthiness** - Techniques to enhance trustworthiness and credibility of data analysis (e.g., member checking, audit trail, triangulation); rationale** | | Pages 4/5 | |
|  | |  | |
| **Results/findings** |  | |  |
| **Synthesis and interpretation** - Main findings (e.g., interpretations, inferences, and themes); might include development of a theory or model, or integration with prior research or theory | | Pages 5 to 8 | |
| **Links to empirical data** - Evidence (e.g., quotes, field notes, text excerpts, photographs) to substantiate analytic findings | | Supplementary Table 1 | |
|  | |  | |
| **Discussion** |  | |  |
| **Integration with prior work, implications, transferability, and contribution(s) to the field -** Short summary of main findings; explanation of how findings and conclusions connect to, support, elaborate on, or challenge conclusions of earlier scholarship; discussion of scope of application/generalizability; identification of unique contribution(s) to scholarship in a discipline or field | | Pages 10/11 | |
| **Limitations** - Trustworthiness and limitations of findings | | Page 11 | |
|  | |  | |
| **Other** |  | |  |
| **Conflicts of interest** - Potential sources of influence or perceived influence on study conduct and conclusions; how these were managed | | Pages 10/11 | |
| **Funding** - Sources of funding and other support; role of funders in data collection, interpretation, and reporting | | Page 1 | |
|  | |  | |
| *The authors created the SRQR by searching the literature to identify guidelines, reporting standards, and critical appraisal criteria for qualitative research; reviewing the reference lists of retrieved sources; and contacting experts to gain feedback. The SRQR aims to improve the transparency of all aspects of qualitative research by providing clear standards for reporting qualitative research. | |  | |
|  | |  | |
| **The rationale should briefly discuss the justification for choosing that theory, approach, method, or technique rather than other options available, the assumptions and limitations implicit in those choices, and how those choices influence study conclusions and transferability. As appropriate, the rationale for several items might be discussed together. | |  | |
|  | |  | |
| **Reference:** | |  | |
| O'Brien BC, Harris IB, Beckman TJ, Reed DA, Cook DA. **Standards for reporting qualitative research: a synthesis of recommendations.** *Academic Medicine*, Vol. 89, No. 9 / Sept 2014  DOI: 10.1097/ACM.0000000000000388 | |  | |
|  | |  | |
|  | |  | |

**Supplementary Figures and Tables**

Supplementary Table S1 - Illustrative quotes supporting themes identified in stakeholder interviews

| **Theme / sub-theme** | **Illustrative quote(s)** |
| --- | --- |
| **Perceived need and scope**  *Value of a dedicated modification reporting tool* | 'I mean, there isn't anything like this currently that exists, there isn't a specific checklist or reporting tool like this … I think it's new and useful. I think it'll be an important addition to the IDEAL framework way of doing things, for sure.' (P2, clinician/methodologist) |
| *Positive reception and anticipated uptake* | 'Yeah, it's good. I like it … I think it would definitely be adopted.' (P6, clinician) |
| **Clarity and content**  *Defining the innovation* | 'When it asks for innovation type, either procedure or device, that's kind of a binary classification. It can be both as well sometimes; some innovations will have the procedure, some will have the device that needs to be installed with a specific procedure associated with it, so it could be both.' (P2, clinician/methodologist) |
| *Patient eligibility criteria wording* | 'It sounds a bit more patient-centred if you phrase it as patient eligibility criteria.' (P2, clinician/methodologist) |
| *Removal of "non-technical modifications"* | 'I can't think off the top of my head what a non-technical modification is because it's either the device or something else related to the other component parts or something about the patient. I don't know.' (P17, methodologist) |
| *IDEAL stage* | 'Using IDEAL as a starting place is really good. It makes people stop and think.' (P8, clinician) |
| **Length and layout**  *Pragmatism and single-page format* | 'Although you want it to be really comprehensive, actually it's got to be completable … I like the way you've kept it to one page.' (P8, clinician) |
| *Avoiding an onerous checklist* | 'You want this to be really pragmatic, because I often find one of the difficulties about all of this stuff is that it just becomes a tick box exercise rather than being meaningful.' (P6, clinician) |
| **Essential components for reporting modifications**  *Reporting of what/when/why* | 'I think that rationale is a very important thing … it's absolutely critical.' (P11, clinician) |
| *Absence of modifications* | 'Yeah, absence of modifications, if no modifications occurred. Yeah, it's really important that you get somebody to positively say that they've not done anything. So 5D is important.' (P8, clinician) |
| *Graphical representations* | 'Consider providing a graphic representation, good.' (P17, methodologist) |
| *Stability of the innovation* | 'I like the idea of the stability of design. It's good.' (P8, clinician)  'No one ever knows what stable means, so this is a good starting point for the stability, just understanding how people are assessing or defining that by asking it as an open question.' (P13, methodologist) |
| *Sharing learning points* | 'Yeah, I think the last point's a good point, that's probably missing at the moment where in the current literature you don't see how the thing's changed, you just get at the end, it's been done on X number of patients, here are the results, whereas they might have learned that doing it in certain ways was a terrible idea, so it's definitely good to have that written down.' (P13, methodologist) |

Supplementary Figure S1 - Timeline of all 20 interviews with significant checklist version updates and key changes annotated

**Supplementary Figure S2**

Illustrative hypothetical example of a completed **SMART checklist**

The following supplementary figure provides an illustrative example of a completed SMART checklist, based on a hypothetical innovation and study. The fictitious example here is an innovative procedure we have called ‘Laparoscopic Preperitoneal Rectus Diastasis Repair (LPRDR)’.

The checklist example below has been completed with page numbers from a hypothetical study and pre-publication manuscript. Below the checklist is example text that corresponds with the checklist items.

| **Section/Topic** | **Item** | **Checklist item** | | | **Where reported** |
| --- | --- | --- | --- | --- | --- |
| **Describe the starting point of the innovation:** | | | | | **Page Number(s):** |
| **Describe the innovation** | 1 | ***Describe the innovation*** (procedure, part of procedure and/or device) as it existed at the ***start*** of the current report/description of use (i.e. before any modifications occur). This should encompass a description of its constituent components and individual steps. Definitions of surgical innovations can be found [here (page 18)](https://www.rcseng.ac.uk/-/media/files/rcs/standards-and-research/standards-and-policy/good-practice-guides/old-documents/surgical-innovation-new-techniques-and-technologies.pdf). | | | *1* |
| **Outline patient eligibility criteria** | 2 | ***Describe the initial patient eligibility criteria*** (i.e. the inclusion and exclusion criteria) used to select which patients received the innovation (before any modifications occurred). | | | *2* |
| **Indicate the IDEAL stage(s)** | 3 | Identify the most appropriate [***IDEAL stages***](https://journals.lww.com/annalsofsurgery/abstract/2019/02000/no_surgical_innovation_without_evaluation_.6.aspx) or [***IDEAL-D stages***](https://www.bmj.com/content/353/bmj.i2372.long) for the reported procedure or device. | | | *2* |
| **Report contextual information** | 4a | Provide an ***overview of the context*** in which the procedure or device was used in the current report in terms of:  ***- Timeframe*** (i.e. dates) being reported | | | *2* |
|  | 4b | ***- Number and type of centres*** involved (e.g. sizes, locations and functionality/specialisation) | | | *2* |
|  | 4c | ***- Number and type of surgeons/operators*** performing the procedure/using the device (e.g. general experience, innovation-specific experience and training, grade, speciality) | | | *2* |
|  | 4d | ***- Number of times innovation was delivered,*** including the number of patients receiving the innovation | | | *2* |
| **List all modifications to the innovation:** | | | **Page number(s):** | | |
| **Report all modifications*** | | | **i. Which patients**† | **ii. Why**‡ | **iii. Implications**§ |
| **Technical** | 5a | For ***procedures***, examples include changes to specific procedural steps (e.g., addition, subtraction, repetition, sequence, timing or exchange), surgical approach (e.g. robotic/laparoscopic), incision/access, patient positioning, devices/tools/adjuncts used for a procedure and periprocedural aspects (e.g. medication, anaesthesia). | *4-5* | *4-5* | *4-5* |
|  |  | For ***devices***, this may include changing device size, increasing or decreasing the number of parts/components, substituting a constituent part for another or altering the number of devices used. It may also include modifications to the procedure in which the device is being used or how a device is used/applied/inserted (i.e. instructions for use). | *N/A* | *N/A* | *N/A* |
| **Patient eligibility criteria** | 5b | Changes to ***inclusion and/or exclusion criteria***, such as changes in complexity, co-morbidities included or pathology treated | *5* | *5* | *5* |
| **Abandoning, stopping or converting** | 5c | Any instances where a decision was made to ***stop using an innovation*** or where it was ***converted*** to another procedure or approach (e.g. robotic to open surgery), either in an individual patient or for all further patients. Here, the alternative device or procedure used should be included. | *5* | *5* | *5* |
| **Absence of modifications** | 5d | If ***no modifications have occurred*** (to aspects covered in 5a-c), report on the absence of modifications. | *N/A* | | |
| **Summarise modifications:** | | | | | **Page Number(s):** |
| **Assess stability** | 6 | Provide views on the ***stability*** of the innovation at the end of the time period being reported (i.e. whether further changes are anticipated or planned). This stability may apply to the entire procedure/device or specific steps/components. | | | *6* |
| **Share learning** | 7 | Summarise any ***learning points*** gained from making modifications. Examples include implications from technical or patient eligibility criteria modifications that others could learn from. | | | *7* |

**To aid with reporting, authors are encouraged to: Report modifications in a* ***dedicated sub-section*** *within the* ***main report*** *rather than supplementary materials. If supplementary materials are necessary, modifications reported within them should be clearly* ***signposted*** *in the main text. Also consider providing a* ***graphic representation*** *to aid reporting, e.g., the timeline of when modifications occurred during the series of procedures being reported/uses of the device*

†**Which patients** – How many patients the modification was used for. ‡ **Why** – The rationale. § **Implications** – Advantages and disadvantages.

The SMART Checklist is copyrighted © 2026 by the University of Bristol Centre for Surgical Research. Users of this tool are kindly requested to cite this original definitive paper: *Olivier JB, Macefield RC, Coyle M, Avery KNL, Blencowe NS, Elliott D. A concise stakeholder-informed tool for reporting modifications to innovative surgical procedures and devices: the SMART checklist. BJS. 2026*

- **Item 1: Describe the innovation**
  - *Completed Example Text in an Introduction section of a manuscript, e.g. page 1:* “Laparoscopic Preperitoneal Rectus Diastasis Repair (LPRDR) involves a three port laparoscopic approach to reconstruct midline fascial laxity. The initial configuration consists of creating a preperitoneal workspace using a dissection balloon, followed by primary plication of the linea alba using a single row of continuous running barbed sutures without mesh reinforcement.”
- **Item 2: Outline patient eligibility criteria**
  - *Completed Example Text in the Methods section, e.g. page 2:* Initial eligibility criteria restricted recruitment to adults with a symptomatic rectus diastasis measuring greater than four centimetres on computed tomography imaging.
- **Item 3: Indicate the IDEAL stage(s)**
  - *Completed Example Text in the Methods section, e.g. page 2:* This study represents an IDEAL Stage 2a Development study.
- **Item 4a: Timeframe**
  - *Completed Example Text in the Methods section, e.g. page 2:* The study period spanned from January 2025 to December 2025.
- **Item 4b: Centres**
  - *Completed Example Text in the Methods section, e.g. page 2:* The study was conducted at a single tertiary referral university teaching hospital.
- **Item 4c: Surgeons**
  - *Completed Example Text in the Methods section, e.g. page 2:* Two consultant upper gastrointestinal surgeons with extensive experience in abdominal wall reconstruction but no prior experience with this specific technique performed all procedures.
- **Item 4d: Delivery volumes**
  - *Completed Example Text in the Methods section, e.g. page 2:* The procedure was delivered 25 times to 25 individual people.
- **Item 5a: Technical modifications** *(Completed Example Text in the Results section, e.g. pages 4-5)*
  - *i. Which patients:* The eleventh patient in the study series received a modification consisting of the inclusion of a mesh within the preperitoneal space and this was continued for all subsequent patients thereafter.
  - *ii. Why:* the modification was introduced due to 2 patients having an early fascial dehiscence.
  - *iii. Implications:* Advantages of the modification included a significant increase in midline repair integrity with no subsequent fascial failures. Disadvantages of the modification included a mean increase in operative duration of 18 minutes.
- **Item 5b: Patient eligibility criteria modifications** *(Completed Example Text in the Results section, e.g. pages 4-5)*
  - *i. Which patients:* After the 16^th^ patient in the study the eligibility criteria were expanded to include patients with BMI up to 33.
  - *ii. Why:* Favourable wound healing and low complication rates observed in the first 15 patients provided reassurance to expand the inclusion criteria.
  - *iii. Implications:* Advantages of modifying the inclusion criteria included wider clinical utility for a broader patient demographic. Disadvantages included increased technical difficulty due to adiposity.
- **Item 5c: Abandoning, stopping or converting** *(Completed Example Text in the Results section, e.g. page 5)*
  - *i. Which patients:* The procedure was converted to open for the 8^th^ patient.
  - *ii. Why:* An inadvertent tear in the peritoneum occurred during the initial retrorectus space creation.
  - *iii. Implications:* This negated the intended benefits of a minimally invasive approach for that patient.
- **Item 6: Assess stability**
  - *(Completed Example Text in the Discussion section, e.g. page 6):* The technical configuration of the repair achieved procedural stability after the 15^th^ patient. The addition of a preperitoneal mesh placement proved reproducible and safe, and no further structural alterations to the technique are planned for future cohorts.
- **Item 7: Share learning**
  - *(Completed Example Text in the Discussion section, e.g. page 6):* Reconstructing a severe rectus diastasis via a primary laparoscopic plication alone carries an unacceptable risk of early fascial attenuation and suture pull through. Innovators utilising this technique should adopt the use of a reinforcing mesh early in their learning curve to ensure mechanical stability and avoid structural failure.

**References**

1.	Olivier J, Elliott D, Avery K, Blencowe NS, Macefield R. Identifying and reporting modifications to surgical innovation: a systematic review of IDEAL/IDEAL-D studies. BMJ Open. 2025;15(6):e097097.

2.	Moher D, Schulz KF, Simera I, Altman DG. Guidance for Developers of Health Research Reporting Guidelines. PLoS Medicine. 2010;7(2):e1000217.

3.	EQUATOR. The EQUATOR (Enhancing the QUAlity and Transparency Of health Research) Network 2025 [Available from: <https://www.equator-network.org/about-us/>.

4.	Schulz KF, Altman DG, Moher D, CONSORT. CONSORT 2010 statement: Updated guidelines for reporting parallel group randomised trials. Int J Surg. 2011;9(8):672-7.

5.	Hossaini S, Hoffmann C, Cousins S, Blencowe N, McNair AGK, Blazeby JM, et al. Development of a conceptual framework for reporting modifications in surgical innovation: scoping review. BJS Open. 2023;7(2).
